# Tunable zero-field ferromagnetic resonance frequency from S to X band in oblique deposited CoFeB thin films

**DOI:** 10.1038/srep17023

**Published:** 2015-11-23

**Authors:** Chengyi Li, Guozhi Chai, Chengcheng Yang, Wenfeng Wang, Desheng Xue

**Affiliations:** 1 Key Laboratory for Magnetism and Magnetic Materials of the Ministry of Education, Lanzhou University, Lanzhou, 730000, People’s Republic of China

## Abstract

Tunable zero-field ferromagnetic resonance frequency in wide range is very useful for the application of microwave devices. We performed an investigation of the static and high frequency magnetic properties for oblique sputtered CoFeB thin films. The static magnetic results revealed that oblique sputtered CoFeB thin films possess well defined in-plane uniaxial magnetic anisotropy, which increases monotonically from 50.1 to 608.8 Oe with the increasing of deposition angle from 10° to 70°. Continuous modification of the resonance frequency of CoFeB thin films in a range of 2.83–9.71 GHz (covers three microwave bands including S, C and X bands) has been achieved. This behavior can be explained as the result of the microstructure due to the self-shadowing effect mainly. These CoFeB thin films with tunable magnetic properties may be good candidates for usage in microwave devices.

Magnetic materials are widely used in many microwave devices, such as magnetic sensors, energy harvesters, phase shifters, tunable filters and so on^1^,^2^,^3^, Compared with bulk materials, magnetic thin films, which have higher in-plane anisotropy, are widely studied in many works^4^,^5^,^6^, Most of the recent works focus on pushing the resonance frequency *f*_*r*_ of films to over 5 GHz, however, it is still a challenge to find a method to push the resonance frequency of thin films to much higher level (for example, X band) and adjust the *f*_*r*_ in a wide frequency range simultaneously^7^,^8^,^9^. Based on the Kittel equation^10^, the zero-field *f*_*r*_ of thin film is dependent on the in-plane effective anisotropy field *H*_*K*_ and the saturation magnetization 4π*M*_*s*_. Thus, it is possible to adjust the resonance frequency by tuning the value of *H*_*K*_. There are several methods to induce in-plane magnetic anisotropy, for example, including applying DC magnetic field during film deposition and/or anneal^11^,^12^, *in situ* depositing on pre-stressed substrates^13^, exchange bias^14^,^15^, multilayer structure^16^,^17^, and oblique deposition^18^,^19^, etc. Each of the above methods has its own features: the value of *H*_*K*_ induced by applying DC magnetic field is relatively hard to modified, the pre-stressed substrates method requires the substrates to be flexible, exchange bias may only tune the anisotropy in ultra-thin films, and multilayer structure change the *H*_*K*_ only in very thin interlayers with interlayer interaction^13^. Thus, oblique deposition is one of the most effective and simplest ways to tune the magnitude of anisotropy field in regular thin films.

The other key parameter that affects the resonance frequency is 4π*M*_*s*_, and the permeability is also proportional to the saturation magnetization. Thus, large 4π*M*_*s*_ is necessary for both higher permeability and higher resonance frequency. CoFe alloy, which is one of the magnetic materials with largest saturation magnetization, is widely investigated in magnetic thin films^20^,^21^,^22^,^23^, Among these works, the Ru/FeCoB double layered film also indicates that large anisotropy can be induced in FeCoB layer^24^, In FeCoB thin films, the element B was selected as dopant to reduce the magneto-crystalline anisotropy, then, the in-plane uniaxial anisotropy of the films can be induced more easily^25^, In this work, we choose Co_69.0_Fe_27.8_B_3.2_ (CFB) as the composition of the magnetic thin films, and combine with the oblique deposition technique to obtain such magnetic thin films with not only ultra-high resonance frequency but also tunable resonance frequency in an ultra-large range.

## Results

### Structure of the oblique deposited CFB magnetic thin films

**Figure 1:**
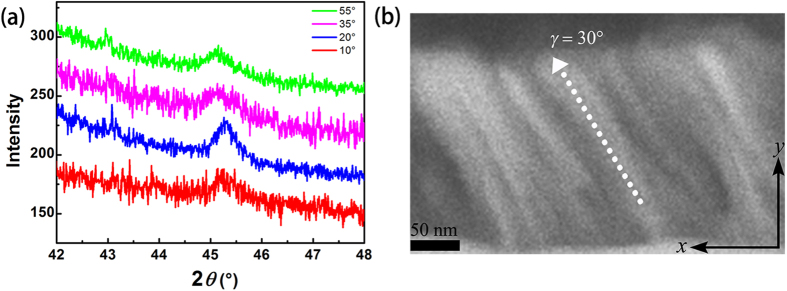
Structures of the oblique deposited CFB films. (**a**) XRD results for CFB thin films with different oblique deposition angle and (**b**) cross-sectional image of CFB thin films deposited at 70 °.

The thickness of the CFB magnetic thin films are all around 40 nm. The XRD spectrum of the oblique deposited CFB thin films is shown in Fig. 1(a). The peak positions of all of the films in the diffraction spectra almost keep the same around 45.20°, which agrees with the (110) diffraction peaks of BCC Co_7_Fe_3_ with Powder Diffraction File (PDF) number 50–0795. This shows that the films are all BCC structure, and the lattice spacing does not changed with the oblique deposition changing. By analyzing the spectra, the full width at half maximum (FWHM) values of different deposition angles can be obtained. After putting the FWHM values into the Scherrer equation^26^, the grain size is calculated with 11.32 nm, which means the CFB thin films are basically nano-crystalline thin films. The small grain size is due to the doping of the B element into the films, which can reduce the grain size during the deposition as described before^25^. The cross-sectional image of CFB thin films deposited at 70° is shown in Fig. 1(b). (As the SEM we utilized can’t observe clearly microstructural image of the cross section of thin films with thickness below 100 nm. Here, we show an image of 200 nm CFB thin film with 70° deposition angle.) It can be seen that the tilted column crystal in the image. The oblique angle of the tilted column crystal is 30° which is smaller than the incidence angle. This result is consistent with the results from other literatures with relation as 2tan*γ* = tan*β*, where *γ* is the angle of tilted column and β is oblique incident angle^27^.

### Static magnetic properties of the oblique deposited CFB magnetic thin films

**Figure 2:**
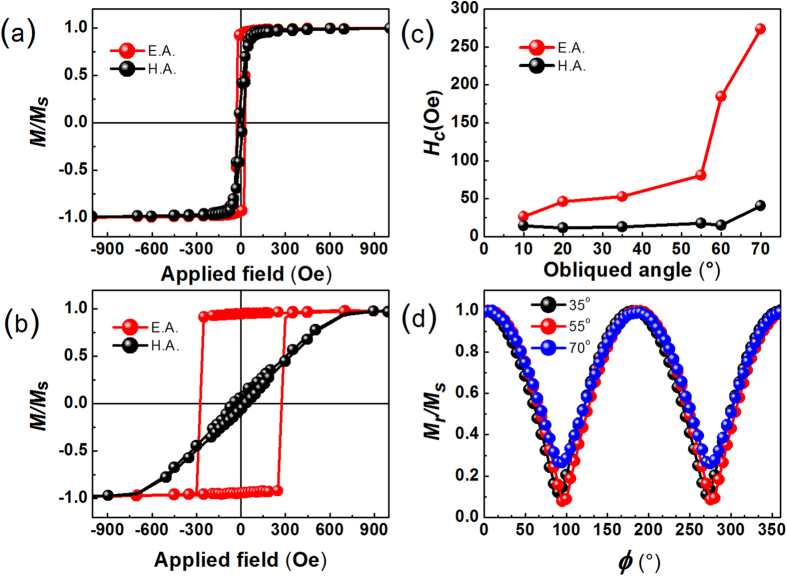
Static magnetic properties of the CFB films. Hysteresis loops of CFB films with the oblique angle of 10° (**a**) and 70° (**b**). EA (HA) means the loops were measured by applying the field in easy axis (hard axis). (**c**) Coercivity of CFB films with different oblique angle. (**d**) In-plane angular dependence of *M*_*r*_/*M*_*s*_ on the angle *φ*, between the field direction and the easy axis of thin film.

**Figure 3:**
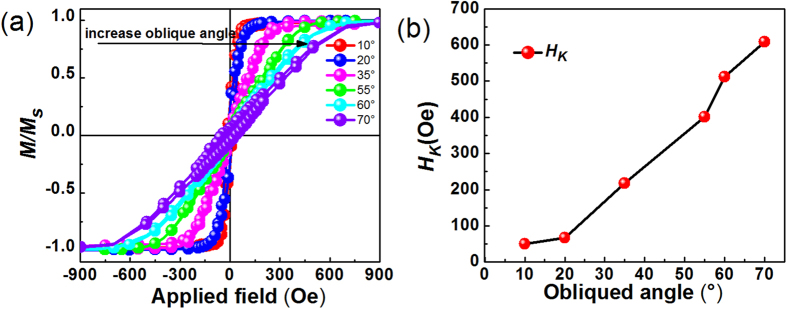
In-plane anisotropy of the CFB films. (**a**) The hard axis Hysteresis loops of CFB thin films deposited in different oblique deposition angles. (**b**) Dependence of *H*_*K*_ on incidence angle.

Figure 2(a,b) show the in-plane magnetic hysteresis loops along easy and hard axis of the thin films deposited with oblique angle 10° and 70°. The 4π*M*_*s*_ is obtained about 18.52 kG by the total saturated magnetic moments and the volume of the thin films. The M-H loop along easy axis looks like a well rectangle shape; meanwhile the hard axis hysteresis loop is basically a closed curve with very small hysteresis. These shapes of magnetic hysteresis loops reveal that these samples were well defined in-plane uniaxial magnetic anisotropy. For uniaxial anisotropy magnetic thin films, the anisotropy field is related to the saturated field on hard axis. From the Fig. 2(a,b), it is easy to find that the saturated field on hard axis of film deposited with oblique angle 70° is much larger than the film deposited with oblique deposition angle 10°.This indicates the anisotropy field of the oblique deposited CFB thin films can be adjusted by changing the deposition angle. Figure 2(c) shows the dependence of coercivity (*H*_*c*_) values vs. the oblique deposited angle. The *H*_c_ along hard axis is lower than 20 Oe for all the CFB thin films. The *H*_*c*_ along easy axis increases with the increasing oblique angle, which is a common result in oblique deposited thin films^28^, Fig. 2(d) shows the remanent magnetization curve (*M*_*r*_*/M*_*s*_) of films with different deposited angles. All the curves were measured from the easy axis at the beginning, thus, 0° and 180° means the value of remanent magnetization along easy axis, 90° and 270° means the value of remanent magnetization along hard axis. The different shapes and colors of the symbols denote the results taken for different oblique deposited angles, which are also marked in the figure. The values of *M*_*r*_*/M*_*s*_ along the easy axis are all approximate 1.0, however, the all values of *M*_*r*_*/M*_*s*_ along the hard axis are very small, which indicates almost all the magnetic moments in CFB thin films are aligned along the easy axis in the demagnetized state due to the well-defined in-plane uniaxial magnetic anisotropy. All the above static magnetic measurements results have revealed that the as-deposited CFB thin films have good soft magnetic properties and possess a well- defined in-plane uniaxial magnetic anisotropy. Figure 3(a) shows the hard axis hysteresis loops of films deposited at various deposition angles. The saturation field of these films is increasing with the increasing of oblique angle, which means that the *H*_*K*_versus oblique angle has the same behavior with the saturation field. After calculating the *H*_*K*_ by calculating the measured area of easy axis and hard axis loops of the reduced magnetization^29^, the *H*_*K*_ dependence on the oblique angles is plotted out in Fig. 3(b) as a summary. The result shows that *H*_*K*_ of the CFB films monotonously increases from 50.1 to 608.8 Oe with the oblique deposition angle increasing from 10 ° to 70°.

### Tunable high frequency properties of the oblique deposited CFB magnetic thin films

**Figure 4:**
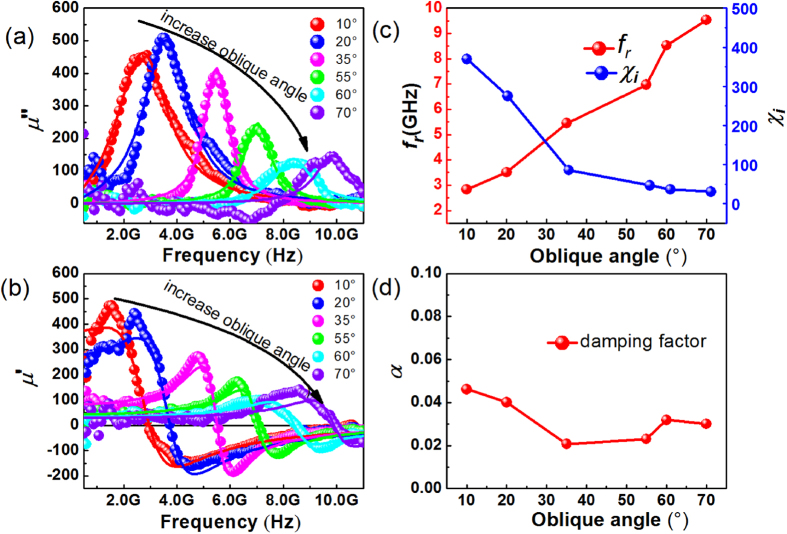
High frequency properties of CFB films. The imaginary (**a**) and real (**b**) parts of high frequency permeability spectra of CFB thin films with different oblique deposition angle. Dependence of the resonance frequency *f*_*r*_ and initial magnetic susceptibility (**c**) and damping factor (**d**) of the CFB thin films with different oblique deposition angle.

Based on the Kittel’s equation, the resonance frequency is highly depended on the in-plane magnetic anisotropy field. As the in-plane magnetic anisotropy field increasing with the oblique deposited angle, the high frequency performance of CFB should also be adjusted by changing the oblique angle. The dynamic permeability spectra of the films deposited under different oblique angles are presented in Fig. 4 (a) for imaginary part and (b) for real part. The different colors of the symbols show the experimental results and the lines with the same color are the fitting curve for the same sample with an expression carried out by procession progress of the magnetic thin films with LLG (Landau-Liftshitz-Gilbert) equation^30^, It is clear to see that the resonance peak of the imaginary part moves to higher frequency when the deposition angle is increasing from 10° to 70°. This means the zero-field ferromagnetic resonance frequency is increasing with the deposition angles change. After fitting the experimental data with theoretical equations, the *f*_*r*_ and the initial magnetic susceptibility *χ*_*i*_ (equal to *μ*_*i*_-1) of the CFB thin films can be obtained. The *f*_*r*_ and *χ*_*i*_ dependence on the oblique angles are shown in Fig. 4(c). The *f*_*r*_ of the CFB magnetic thin films increases significantly from 2.83 GHz to 9.71 GHz with the oblique deposition angle increasing from 10° to 70°. This result also reflected the change of in-plane uniaxial magnetic anisotropy of the CFB film. The initial magnetic susceptibility is reducing from 369.6 to 30.4 with the oblique angle increasing. This result can be explained by Snoek’s law that the permeability and the resonance frequency show a trade-off behavior^31^, The damping coefficient *α* vs oblique deposition angle is shown in Fig. 4(d). The damping factor is decreasing from 0.046 to around 0.02 with different oblique angles, and it is consistent with the change of line width of permeability spectra.

## Discussions

**Figure 5:**
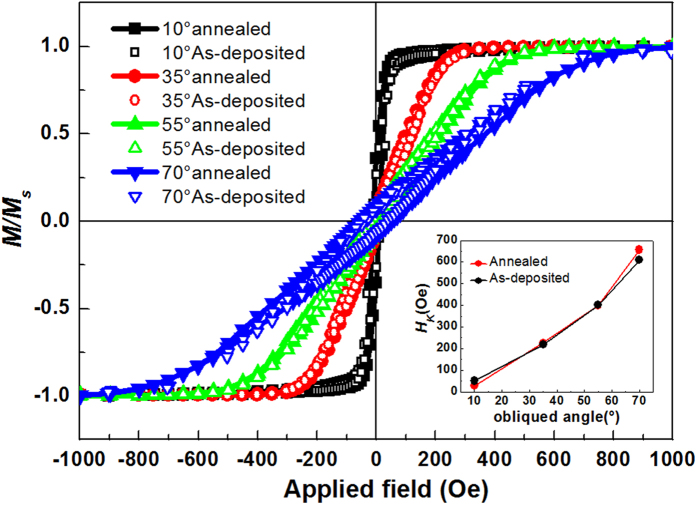
Static magnetic properties of the annealed compared with as-deposited CFB films. Hysteresis loops of annealed and as-deposited CFB films with oblique angle of 10°, 35°, 55° and 70°. Insert is the comparison of the in-plane anisotropy of the corresponding films.

Based on previous reports, two possible effects may contribute to the in-plane uniaxial magnetic anisotropy in oblique deposited thin films: (1)anisotropic stress in combination with the isotropic magnetostriction^32^, and (2) the column chain structure developed in the film plane due to the so-called self-shadowing effect^32^,^33^, As annealing process can eliminate residual stresses by thermal motion of the atoms^34^,^35^,^36^, we measure the loops of these thin films with oblique deposition 10°, 35°, 55°and 70°after annealing. The Hysteresis loops of these annealed CoFeB thin films are shown in Fig. 5 and the insert is the *H*_*K*_ dependence on the oblique angles. The loops have no evident difference compared with loops of as-deposited films. From the inserted figure, the anisotropies of annealed films are also consistent with the as-deposited. It is indicated the strain has less effect on the anisotropy field. From the cross-sectional images showed in Fig. 1(b) above, it is a reasonable conjecture that the in-plane uniaxial magnetic anisotropy is mainly the result of column chain structure developed in the film plane due to the self-shadowing effect^37^, For damping coefficients *α*, it includes two parts, one is the intrinsic damping and the other is extrinsic damping. The extrinsic damping is related to the dispersion of the magnetic moment in remanent state. In magnetic thin films, larger uniaxial anisotropy can make more magnetic moment align in easy axis of the anisotropy. Thus, the extrinsic damping reduces due to the dispersion of the magnetic moment decreased. The total damping of the magnetic thin films decreases with the oblique deposited angle increasing.

In summary, we have performed an investigation of the influence of the oblique deposition angle on the static and microwave magnetic properties of CFB thin films. It is found that the oblique deposition angle can tailor the resonance anisotropy significantly from 2.83 GHz to 9.71 GHz, which covers three microwave bands including C, S, and X bands. The origin of the in-plane uniaxial magnetic anisotropy is mainly due to the self-shadowing effect arising from the formation of tilted columnar structure in oblique deposited films. These magnetic thin films with resonance frequency that can be tuned in such large range should be useful for the application in microwave devices.

## Methods

### Samples preparation

**Figure 6:**
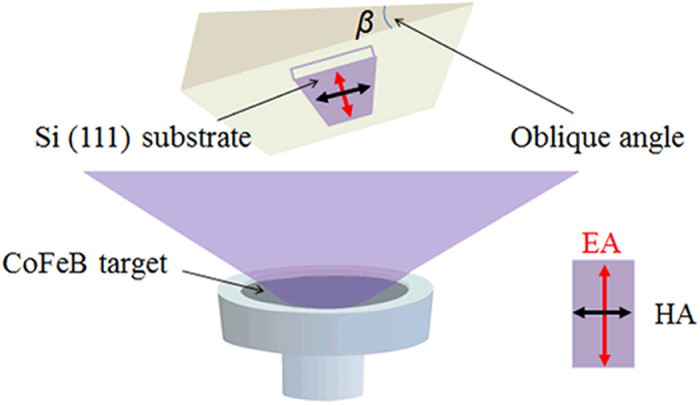
Schematic diagram of the sputtering arrangement. A FeCoB target was used to deposit the thin films. The Si was place on the upholder with different incident angle from 10° to 70°.Easy axis of the films are marked with the red arrow.

A radio frequency (RF) magnetron sputtering chamber was used to deposit 40-nm thick Co_69.0_Fe_27.8_B_3.2_ magnetic thin films onto 10 × 20 × 0.42 mm^3^ Si(111) substrates at ambient temperature with background pressure lower than 5 × 10^−5^ Pa. A 2-inch CoFeB target with atom ratio as 67:29:4 was placed onto the target-mounting surface of the target gun to deposit the films. During the sputtering, the RF power density was 2.47 W/cm^2^. The thickness of the thin film was controlled by both the deposition time and a constant deposition rate. These films were deposited with oblique incidence angles from 10° to 70 ° to induce different uniaxial anisotropy. Ar gas flow rate of 20 SCCM (SCCM denotes cubic centimeter per minute at STP) was kept constant to maintain an Ar pressure of 0.3 Pa during sputtering. We show the schematic diagram of oblique sputtering method in Fig. 6.

### Measurement

The crystallographic of the films was characterized by X-ray diffraction (XRD, X’Pert PRO PHILIPS with Cu K_α_ radiation). A field emission scanning electron microscope (SEM, Hitachi S-4800) was used to obtain the cross section image. The thicknesses of the magnetic thin films are also obtained from the cross section images. The in-plane magnetic hysteresis loops of the films were measured at room temperature by using a vibrating sample magnetometer (VSM, Lakeshore 7304 model) and the saturated magnetization can be obtained by the division of all saturated magnetic moment and film volume. The value of *H*_*K*_ was obtained from the loops by calculating the measured area of easy axis and hard axis loops of the reduced magnetization^29^, which is an effective method to calculate *H*_K_ for thin films with uniaxial in-plane magnetic anisotropy. The permeability spectra was carried out with a PNA E8363B vector network analyzer by using one port shorted microstrip method from 100 MHz to 11 GHz^38^, The *f*_*r*_ and damping parameter *α* were determined by fitting the permeability spectrum with theoretical equation derived from Landau-Lifshitz-Gilbert (LLG) equation^30^,^39^, All the above observations and measurements were performed on as-deposited samples at room temperature.

## Additional Information

**How to cite this article**: Li, C. *et al.* Tunable zero-field ferromagnetic resonance frequency from S to X band in oblique deposited CoFeB thin films. *Sci. Rep.* **5**, 17023; doi: 10.1038/srep17023 (2015).
